# Systemic Lupus Erythematosus Complicated with Hypertrophic Cardiomyopathy: A Case Report and Literature Review

**DOI:** 10.1155/2021/6633085

**Published:** 2021-04-11

**Authors:** Huihui Ma, Xin Cao, Jing Zhang, Yongmei Zhou, Rong Luo, Tao He, Jianhong Tao, Xiaoping Li

**Affiliations:** ^1^Department of Cardiology, Sichuan Provincial People's Hospital, University of Electronic Science and Technology of China, Chengdu, Sichuan 610072, China; ^2^Chinese Academy of Sciences Sichuan Translational Medicine Research Hospital, Chengdu 610072, China; ^3^Chengdu University of Traditional Chinese Medicine, Chengdu, Sichuan 610075, China; ^4^Institute of Geriatric Cardiovascular Disease, Chengdu Medical College, Chengdu, Sichuan 610500, China

## Abstract

A 32-year-old female with systemic lupus erythematosus (SLE) for more than 7 years, and long-term treatment with cyclophosphamide, cyclosporine, methotrexate, and tacrolimus, later found to be combined with hypertrophic cardiomyopathy (HCM) for one year. The patient denied a family history of cardiomyopathy and sudden cardiac death (SCD). Echocardiography suggested that uneven thickening of the left ventricle (LV), mainly in the lower middle segment. Cardiac magnetic resonance (CMR) showed that the walls of the left ventricular (LV) were significantly thickened, as about 21 mm, mainly in the middle and lower segments. Genetic tests showed no known or suspected pathogenic variations were found and no significant enhancement in CMR, so secondary HCM was diagnosed clinically. After symptomatic treatment, the patient was discharged, and long-term follow-up was conducted. The diagnosis of HCM, which combined with SLE or second to usage of tacrolimus, was based on symptoms, echocardiography, and CMR; no endomyocardial biopsies were performed.

## 1. Introduction

Hypertrophic cardiomyopathy (HCM) is a disease characterized by cardiac hypertrophy, which is usually manifested as left ventricular hypertrophy [1, 2]. HCM can be diagnosed by left ventricular wall or septal thickness ≥ 15 mm measured by two-dimensional echocardiography or ≥13 mm in patients with a definite family history [1–4]. Systemic lupus erythematosus (SLE) is an autoimmune disease, and the etiology is not completely clear. It is characterized by autoantibodies and immune complexes, which can cause multiple system damage [5]. Cardiovascular system is one of the most frequently involved systems, and cardiovascular events are also an important cause of death in lupus patients [6–12]. Up to 9% of patients with SLE are reported to have cardiac manifestations, including myocarditis, cardiomyopathy (CM), and/or heart failure (HF) [13]. Whether there is cardiomyopathy directly caused by lupus is not clear [13]. We present the 10th case of HCM associated with SLE and discuss the possible causes of cardiac hypertrophy in this patient.

## 2. Clinical Data

### 2.1. Case Report

A 32-year-old female was admitted to the emergency department for 6-day history of fever accompanied by abdominal pain and diarrhea. The highest body temperature was about 39.4°C, with obvious chill and Raynaud's phenomenon. The local clinic provided anti-inflammatory, antiviral, and antipyretic treatments such as “Cephalosporins, Ribavirin and Lysine Acetylsalicylate”, but the body temperature was not significantly decreased. After that, the patient continued to have fever; the body temperature was monitored at 37.8-38.5°C, so she was admitted to our emergency department.

The patient was diagnosed with SLE for more than 7 years. After that, she was hospitalized in our hospital for several times due to recurrent fever accompanied by abdominal pain, diarrhea, nausea, vomiting, etc., and received anti-infection and immune regulation treatment (long-term use of cyclophosphamide, cyclosporine, methotrexate, and tacrolimus).

### 2.2. Physical and Laboratory Examination

After admission, the patient presented with Cushing's syndrome. Bone friction was positive, and there was no cardiac murmur.

Laboratory evaluations revealed the following: leukocyte count was 7.160 × 10^9^/L, neutrophil count was 6.480 × 10^9^/L, platelet count was 188 × 10^9^/L, and hemoglobin level was 131 g/L. Brain natriuretic peptide (BNP) was 752 pg/mL. Procalcitonin was 0.08 ng/mL. Erythrocyte sedimentation rate was 27 mm/h. Carbohydrate antigen 19-9 (CA19-9) was 63.86 U/nL. Complement C3 was 0.313 g/L; complement C4 was 0.078 g/L. The results of anti-Nrnp/Sm antibody, anti-dsDNA antibody, and anti-nucleosome antibody (ANUA) were all positive (+++). Anti-histone antibody (AHA) was also postive (++), and meanwhile, anti-ribosome P-protein antibody (ARPA) and anti-nuclear antibody (ANA) were postive (+), too. Nuclear homogeneous type was 1 : 3200, and nuclear particle type was 1 : 1000.

### 2.3. Electrocardiogram

**Figure 1:**
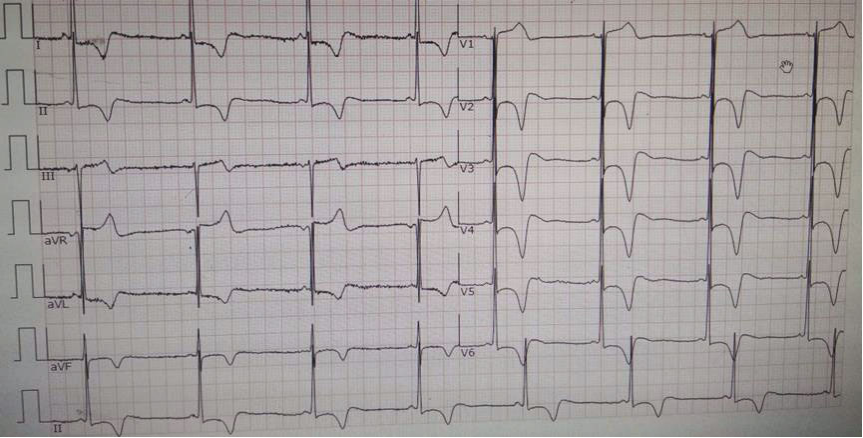
Electrocardiogram showing significant sinus bradycardia, with normal electric axis, short PR interval, high left ventricular voltage, and ST-T change; V2-V6 leads T-wave inversion.

The electrocardiogram of the patient showed significant sinus bradycardia, with normal electrical axis, short PR interval, high left ventricular voltage, and ST-T change. V2-V6 leads T-wave inversion (Figure 1).

### 2.4. Echocardiography

**Figure 2:**
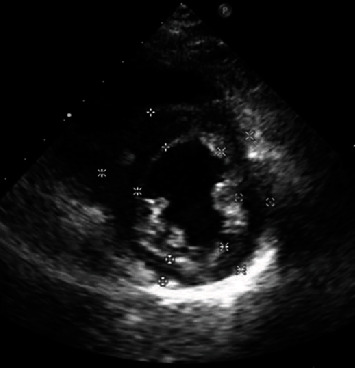
Echocardiography showing the thickened walls in the middle and lower segments.

The echocardiography showed left ventricular diastolic dysfunction, and the left ventricular ejection fraction (LVEF) of the heart was 67%. The cardiac diameter was normal (left atrial, LA = 25 mm; left ventricle, LV = 45 mm; right ventricle, RV = 18 mm; right atrial, RA = 34 × 27 mm). The left ventricular myocardium was unevenly thickened, especially in the middle and lower segments up to 15 mm. Left ventricular diastolic function decreased, E/A = 0.9. Systolic anterior motion (SAM) syndrome was slightly positive. The systolic and diastolic pulmonary blood pressures were about 24 mmHg and 11 mmHg, respectively, with an average of about 16 mmHg (Figure 2).

### 2.5. CMR

**Figure 3:**
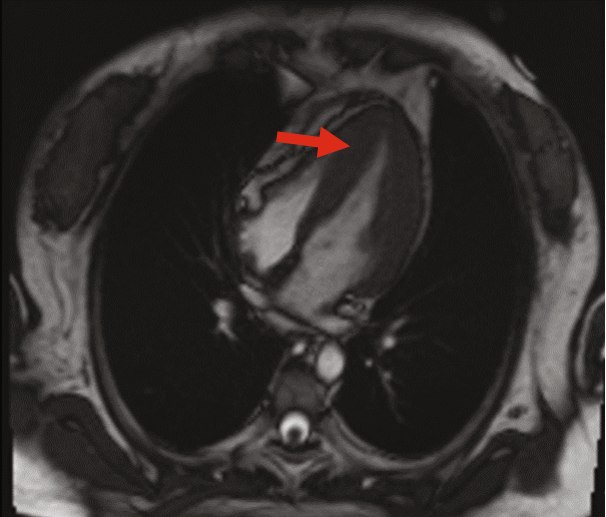
Cardiac magnetic resonance showing the walls of the left ventricle were significantly thickened, mainly in the middle and lower segments (T2-weighted image).

CMR confirmed left ventricular diastolic dysfunction. The walls of the left ventricle were significantly thickened, mainly in the middle and lower segments. The diastolic period thickness was about 21 mm. There was no obstruction in the LVOT and late gadolinium enhancement (LGE) of the myocardial wall. No obvious thickening of the pericardium and effusion was found. On the basis of these data, HCM was diagnosed (Figure 3).

## 3. Genetic Testing

To exclude primary HCM, we used a blood DNA extraction kit to extract genomic DNA from peripheral blood samples. We targeted and enriched for the exons and neighboring introns (within 50 bp) of the 130 genes associated with cardiomyopathy. Based on the guideline for the interpretation of sequence variants: a joint consensus recommendation of the American College of Medical Genetics and Genomics and the Association for Molecular Pathology [14], no known or suspected pathogenic variants consistent with the phenotype were found.

## 4. Outcome and Follow-Up

**Figure 4:**
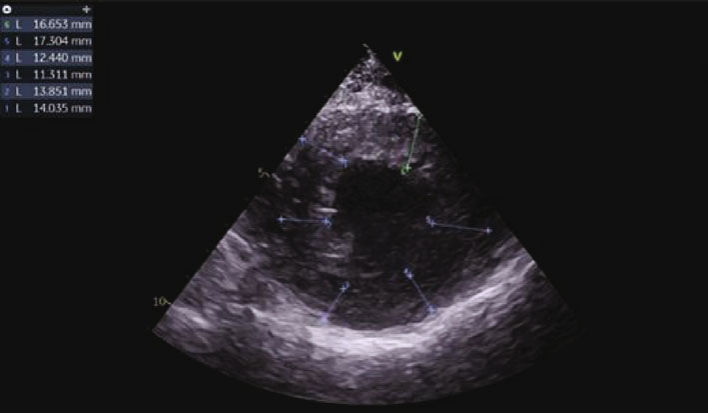
Echocardiography on reexamination showing little thickening of the left ventricular wall.

**Figure 5:**
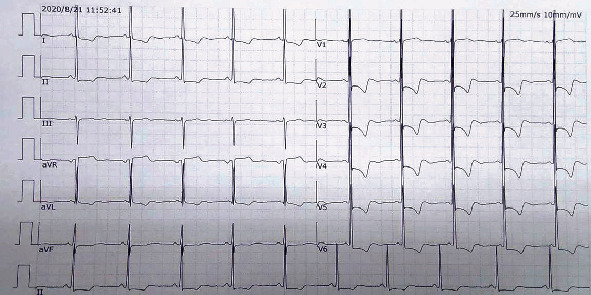
Electrocardiogram on reexamination showing sinus rhythm; T-wave inversion was significantly improved than before.

At a follow-up visit in August 2020, the patient described occasional chest tightness, but the symptoms were not apparent, and the drug was not taken. Echocardiography showed no significant change in ventricular wall thickness from that recorded 1 year earlier (Figure 4), but ECG showed that the T-wave inversion of the V2-V6 leads improved (Figure 5).

## 5. Discussion

We have described a patient with SLE in combination with HCM. The patient had a 7-year history of SLE but no history of myocardial hypertrophy in the previous 6 years. Only nine cases of SLE with HCM have been described, and the underlying mechanisms linking them, if present, are unknown. Therefore, we investigated whether our patient had SLE coincident with primary HCM or HCM secondary to SLE, drug treatment, or unknown cause.

The majority of HCM cases are caused by gene mutations, but it can also occur secondary to other diseases or to long-term use of certain drugs, such as anabolic steroids, tacrolimus, and hydroxychloroquine [15–17]. SLE is a multisystem connective tissue disease that can affect all organs of the body. More than 50% of SLE patients have cardiac involvement, and lesions can involve the pericardium, myocardium, endocardium, valve, conduction system, and coronary arteries [8]. The pericardium is commonly the first site to be involved and occurs in 11%–54% of cases according to electrocardiographic studies [9]. Autopsy findings suggest that about 62% of SLE patients have pericardium involvement [10]. Myocarditis is the most typical feature of myocardial involvement in SLE, and it can progress to ventricular dysfunction, dilated cardiomyopathy (DCM), and HF [11, 12]. Coronary heart disease (CAD) is the most common cause of death in patients with late-onset or long-term SLE [11–13].

The patient described here had received a diagnosis of SLE 7 years before presentation, but she had no family history of HCM or SCD and denied a history of hypertension. Echocardiography and CMR imaging supported a diagnosis of nonobstructive HCM. To exclude idiopathic HCM, we performed next-generation sequencing and analysis with a focus on 130 genes known to be associated with cardiomyopathy. The patient had no known or suspected pathogenic variants consistent with the phenotype, which exclude mostly idiopathic cardiomyopathy and instead suggested HCM secondary to another cause(s).

As noted, the cardiovascular system is commonly involved in SLE [9–12], which initially suggested that SLE may have been the direct cause of HCM. However, HCM has not been clearly linked to SLE to date, and cases of SLE complicated with HCM are rare (Table 1) [18–23]. Among the nine reported cases, eight (89%) were female, and two reported a family history of SCD (*n* = 1) and HCM (*n* = 1) [18, 20]. As shown in Table 1, the patients were relatively young (mean age 36.0 ± 10.4 years) and had a history of systemic disease for many years. HCM was mainly diagnosed by echocardiography, with only one patient undergoing echocardiography and pathological biopsy [23]. The findings of the present study are similar to the previously reported cases. Of note, both SLE and SLE with HCM predominantly affect women.

We considered whether our patient's long-term use of medications such as tacrolimus might be the cause of HCM, especially because her echocardiography and ECG were normal prior to 2019. Diffuse thickening is a key characteristic of drug-induced cardiomyopathy, but it can also manifest as heterogeneous thickening [17, 24], as may have been the case with our patient. Tacrolimus is an effective immunosuppressant most commonly used in patients with kidney, liver, or heart transplants [25]. However, tacrolimus has been reported to have toxic side effects, including cardiotoxicity [17, 26]. Tacrolimus-induced HCM has been described mainly, but not exclusively, in children [11, 27, 28]. And the change is reversible [17]. During follow-up, the patient studied here ceased to take tacrolimus, and although echocardiography showed no significant change in wall thickness, the T-wave inversion of the ECG leads was improved. The patient had no family history of hypertension, HCM, or other cardiovascular disease. Taking these observations into account, we suspected that HCM in our patient was most likely caused by tacrolimus. However, longer follow-up may be needed to prove it.

The goal of this report is to provide further information about the clinical presentation of SLE with HCM and to provide some clues for the study of the relationship between them. Although the concomitant occurrence of the two pathologies may be purely coincidental, advances in molecular biology techniques may shed light on the direct underlying mechanisms linking SLE and HCM.

## 6. Conclusion

To the best of our knowledge, our patient is the 10th reported case of SLE combined with HCM. The specific mechanism linking SLE and HCM is not yet clear, but a significant gender bias is present, with 89% of the reported cases being female. Patients with SLE should be cautioned about the potential for cardiovascular complications.

## Figures and Tables

**Table 1:** Case report about patients with SLE and HCM.

Cases	Age (years)	Gender	Symptom	Drugs	Diagnostic methods (HCM)	References
Asherson et al., 1992	51	Female	Palpitations, chest pain	Steroids, immunosuppressant, warfarin, antidepressant	Echocardiography	[18]
Asherson et al., 1992	45	Female	Palpitations, dyspnea	Propranolol, mexiletine, digoxin, amiodarone	Echocardiography	[18]
Ara et al., 1998	45	Female	Intermittent palpitations, grade II dyspnea, orthopnoea	Hydroxychloroquine	Echocardiography	[19]
Dongji and Yuan, 1998	35	Female	Chest distress, syncope	Hormone, *β*-blocker, calcium antagonist	Echocardiography	[20]
Anastasiadis et al., 2001	32	Female	Arthralgias, malar rush, intermittent palpitations membranoproliferative nephritis	N	Echocardiography	[21]
Anastasiadis et al., 2001	37	Female	Physical and laboratory findings similar to those found in case 1	N	Echocardiography	[21]
Anastasiadis et al., 2001	19	Male	Liver failure due to Budd-Chiari syndrome antiphospholipid syndrome	N	Echocardiography	[21]
Maezawa Linghua, 2002	23	Female	Palpitations	Steroid		[22]
Kotani et al., 2005	37	Female	Exertional chest pain nephrotic syndrome	Prednisolone, cyclophosphamide, mizoribine, *β*-blocker	Echocardiography, pathological biopsy	[23]
The present study	32	Female	Fever accompanied by abdominal pain and diarrhea	Cyclophosphamide, cyclosporin, methotrexate tacrolimus	Echocardiography, CMR	

N: not mentioned in the article; CMR: cardiac magnetic resonance.

## Data Availability

Data are available from the authors upon request. The data that support the findings of this study are available from the corresponding author (Xiaoping Li. E-mail: lixiaoping0119@163.com) upon request.
